# Predictive Utility of the Vedolizumab Clinical Decision Support Tool in a Real-World IBD Cohort: Differential Performance in Crohn’s Disease and Ulcerative Colitis

**DOI:** 10.3390/medicina62040722

**Published:** 2026-04-10

**Authors:** Andreja Ocepek, Nikolaus Molinari, Petra Maček, Jan Zmazek, Sara Nikolić

**Affiliations:** 1 Department of gastroenterology, University Medical Centre Maribor, Ljubljanska 5, 2000 Maribor, Slovenia; 2 Medical Faculty, University of Maribor, Taborska 8, 2000 Maribor, Sloveniapetra.macek99@gmail.com (P.M.); 3 Faculty of Natural Sciences and Mathematics, University of Maribor, Koroška 160, 2000 Maribor, Slovenia

**Keywords:** Crohn’s disease, ulcerative colitis, vedolizumab, clinical decision support tool

## Abstract

*Background and Objectives:* The vedolizumab clinical decision support tool (VDZ-CDST) was developed to predict treatment outcomes in inflammatory bowel disease (IBD). While validated in clinical trial and consortium settings, its real-world performance remains less clear. The aim of our study was to evaluate the predictive value of pre-treatment CDST stratification for clinical and endoscopic outcomes and treatment persistence in real-world VDZ-treated IBD patients. *Materials and Methods:* We conducted a retrospective analysis of consecutive IBD patients initiating vedolizumab therapy, stratified by CDST risk groups. Clinical remission (CR) and corticosteroid-free remission (CSFR) at weeks 14 and 52 were assessed using PRO-2 in both Crohn’s disease (CD) and ulcerative colitis (UC). Endoscopic outcomes and treatment persistence were also evaluated. *Results:* 129 IBD patients, 57 with CD and 72 with UC, treated with vedolizumab were retrospectively stratified according to VDZ-CDST. In CD at week 52 the differences in CSFR between CDST groups were statistically significant (*p* = 0.04). A statistically significant association (*p* < 0.001) was also observed between CDST groups and endoscopic activity (EA) at follow-up endoscopy. In the low-probability group 69.2% showed persistent EA, whereas in the high-probability group 68.8% achieved endoscopic remission (ER). We also found significant differences (*p* = 0.004 and *p* < 0.001, respectively) in treatment persistence between CDST groups in CD. VDZ discontinuation rates were 76.9%, 28.6%, and 6.3% in the low-, intermediate-, and high-response groups, respectively. In UC, no predictive association was observed for either clinical or endoscopic outcomes nor treatment persistence; however, we observed relatively high remission rates despite CDST-based stratification. *Conclusions:* Although the VDZ-CDST failed to predict CR measured by PRO-2 in real-world IBD patients, it demonstrated meaningful associations with long-term CSFR, endoscopic outcomes and treatment persistence in Crohn’s disease. These findings support its role as a supportive tool in therapeutic decision-making, particularly when objective outcomes such as mucosal healing are prioritized. Prospective multicentre studies incorporating biomarkers and pharmacokinetic data are needed to refine VDZ-CDST for broader clinical application.

## 1. Introduction

Inflammatory bowel diseases (IBD), primarily comprising Crohn’s disease (CD) and ulcerative colitis (UC), are immune-mediated conditions characterized by chronic intestinal inflammation in genetically predisposed individuals [1]. Management strategies predominantly involve pharmacological therapy, with surgical intervention reserved for cases complicated by penetrating, structuring, or neoplastic processes [1,2]. Beyond symptom alleviation, the therapeutic objective increasingly emphasizes mucosal healing, which is associated with improved long-term outcomes and decreased complication rates. Early histological and endoscopic control of inflammation has the potential to modify disease trajectory and enhance patient prognosis [3,4]. The advent of advanced biologic therapies has augmented the therapeutic landscape, necessitating tools for optimal patient stratification [5,6]. Identifying patients likely to respond to specific treatments prior to initiation could improve individualized management, reduce adverse effects, decrease healthcare costs, and facilitate adherence to evidence-based algorithms [7]. Current clinical decision-making is supported by guidelines from professional societies, which integrate evidence from clinical research into practice recommendations. Nevertheless, ultimate treatment choices rely on clinician judgement. To assist this process, various scoring systems and predictive tools are being developed, including the Clinical Decision Support Tool (CDST) specifically designed for vedolizumab (VDZ) [8,9,10]. VDZ is a humanized monoclonal IgG-1 antibody that selectively inhibits the interaction between the integrin α4β7 and MAdCAM-1, thereby reducing lymphocyte trafficking to the gut and mitigating inflammation [11]. The CDST for VDZ was initially developed and validated for CD by Dulai et al., who constructed a model to predict response at 26 weeks based on five clinical variables: absence of prior bowel surgery, no exposure to TNF-α inhibitors, absence of fistulizing disease, baseline albumin, and baseline CRP [10]. Patients were stratified into probability groups (low, intermediate, high), with higher response rates observed in the high-probability group, including increased rates of clinical remission and mucosal healing. Subsequently, Dulai et al. adapted and validated a similar CDST for UC, incorporating factors such as prior TNF-α inhibitor exposure, disease duration ≥2 years, baseline endoscopic activity, and albumin levels [9]. Additionally, both models aimed to predict VDZ efficacy and identify patients most likely to benefit from dose optimization [9,12].

The objective of this study was to evaluate the predictive value of pre-treatment stratification using the CDST in a real-world cohort of IBD patients treated with VDZ, focusing on clinical and endoscopic outcomes in line with STRIDE II criteria as well as treatment persistence [13].

## 2. Materials and Methods

### 2.1. Study Design and Setting

This retrospective, single-centre cohort study was conducted at the IBD outpatient clinic of the Department of Gastroenterology, University Medical Centre Maribor, Slovenia, encompassing patients treated from July 2016 to April 2023. The study adhered to the Strengthening the Reporting of Observational Studies in Epidemiology (STROBE) guidelines (Supplementary Materials).

### 2.2. Participants

**Figure 1:**
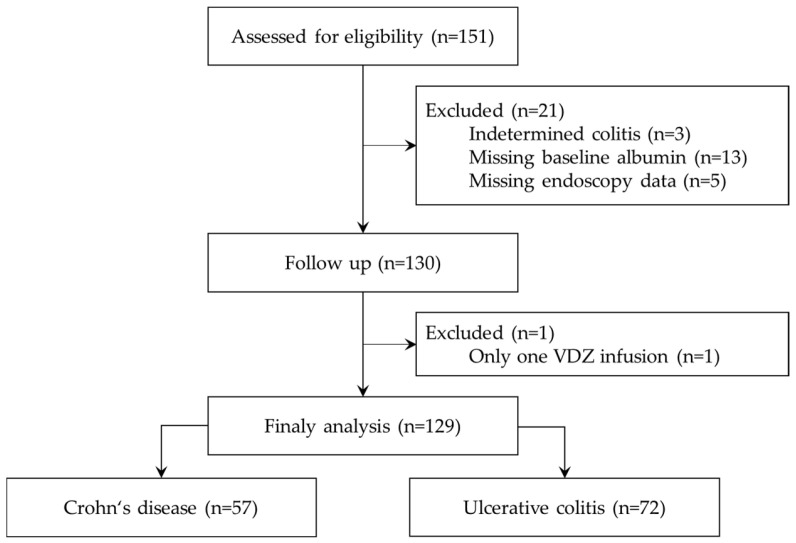
Patient selection flowchart. VDZ = vedolizumab.

Inclusion criteria comprised adult patients diagnosed with CD or UC who received VDZ during the study period. Exclusion criteria included indetermined colitis, age below 18 at first VDZ infusion, follow-up duration less than six months, and missing relevant data. The final study population comprised 129 patients (Figure 1).

### 2.3. Data Collection

Demographic, clinical, and laboratory data were extracted from the UR-CARE registry, including age at diagnosis, gender, disease duration, Montreal classification phenotype, prior surgeries, treatment history (notably TNF-α antagonists), baseline albumin and CRP levels (within three months prior to VDZ initiation), disease activity assessments, concomitant corticosteroid and immunomodulator use, follow-up duration, and baseline endoscopic findings. Due to the nature of retrospective collection, implementation of endoscopic score was not possible in CD; thus, we classified it pragmatically to active disease with ulcers, active disease with erosions, remission. For UC, endoscopic Mayo score was available. All endoscopic data were re-evaluated by the first author, who was blinded to stratification groups.

### 2.4. Exposure and Stratification

Exposure was defined as the response probability group according to CDST (low, intermediate, high). Patients were stratified based on the VDZ CDST scores (Table 1 and Table 2): In the CD response probability groups—low (≤13 points), intermediate (14–19 points), high (>19 points)—were calculated using five variables: no prior bowel surgery, no prior TNF-α inhibitor exposure, absence of fistulizing disease, baseline albumin, and CRP (Table 1) [10]. In the UC response probability groups—low (≤26 points), intermediate (27–32 points), high (>32 points)—were based on variables: absence of prior TNF-α inhibitor exposure, disease duration ≥2 years, moderate endoscopic activity, and baseline albumin (Table 2) [9].

### 2.5. Outcome Measures

Primary endpoints included clinical remission (CR) and corticosteroid-free remission (CSFR) at week 14 and week 52. CR was defined using Patient-Reported Outcomes (PRO) scores: in CD, PRO-2 ≤ 4; in UC, PRO-2 ≤ 1. Secondary outcomes encompassed endoscopic activity at follow-up endoscopy (9–12 months after VDZ initiation in UC and 12–15 months in CD) and treatment persistence assessed over the follow-up period.

Endoscopic outcomes were intentionally defined as secondary, as they reflect a different aspect of disease activity and, particularly in CD, may not correlate fully with clinical outcomes. Endoscopic images were retrospectively reviewed by the first author, who was blinded to CDST risk stratification groups. Standardized endoscopic scores (e.g., SES-CD) were not consistently available in routine clinical documentation, particularly in CD. Therefore, endoscopic outcomes were categorized pragmatically as activity, improvement, or remission based on retrospective image review and available endoscopy reports. Endoscopic activity (EA) was defined as no change from baseline endoscopy. Endoscopic improvement (EI) was defined as ≥50% improvement in CD or a decrease in the endoscopic Mayo score of ≥1 in UC, while endoscopic remission (ER) was defined as absence of ulcers in CD and an endoscopic Mayo score ≤1 in UC. Outcome assessment time points were selected to reflect STRIDE II treatment targets routinely applied in clinical practice at our centre.

Treatment persistence was defined as the time from VDZ initiation to treatment discontinuation for any reason. Patients receiving dose optimization or interval shortening remained classified as persistent on therapy.

### 2.6. Missing Data

After applying the exclusion criteria, 18/151 patients (11.9%) were excluded because of missing data relevant to exposure stratification or outcome assessment. To evaluate the potential for selection bias, baseline characteristics of excluded and included patients were compared separately for UC and CD and are presented in Supplementary Table S1. No relevant differences in baseline characteristics were observed between the groups.

### 2.7. Ethical Considerations

The study was based on the UR-CARE registry data approved by National Ethics Committee 0120-576/2019/7.

### 2.8. Statistical Analysis

Continuous variables were expressed as median with interquartile range (IQR) and categorical variables as percentages. Group comparisons were performed using non-parametric tests: chi-square or Fisher’s exact test for categorical variables, and the Mann–Whitney U test or Kruskal–Wallis test for continuous variables. Treatment persistence at week 52 was evaluated using Kaplan–Meier survival analysis, stratified by CDST groups for CD and UC. In the Kaplan–Meier analysis, treatment discontinuation was considered the event, whereas patients still receiving VDZ at the last available follow-up were treated as right-censored observations. After excluding patients with missing data on key exposure or outcome variables, no further missing data were identified. Statistical analyses were conducted using Jamovi 2.5.4 and Python 3.12. A two-sided *p*-value < 0.05 was considered statistically significant.

## 3. Results

### 3.1. Baseline Characteristic

The study cohort included 129 patients, 57 (44%) with CD and 72 (56%) with UC. The median age was 33.9 years for CD and 32.1 years for UC. Disease duration was longer in CD (median 14.2 years) compared to UC (median 7.9 years). Duration of follow-up while on VDZ treatment was 4.7 years for CD and 3.2 years for UC. Most CD patients had ileo-colonic involvement, with a substantial proportion displaying fistulizing disease and previous surgeries. At baseline, 14% of patients with CD were receiving concomitant corticosteroids and 7% were treated with immunomodulators. Among patients with UC, nearly two-thirds had extensive disease; 34.7% were receiving corticosteroids and 11.1% immunomodulators at baseline. More than half of both CD and UC patients had prior exposure to TNF-α inhibitors. Only two CD patients (3.5%) had been previously treated with ustekinumab, while three UC patients (4.2%) had prior exposure to a Janus kinase inhibitor before initiation of vedolizumab. Baseline endoscopy demonstrated active disease in the majority of CD patients, with 69.8% presenting with deep ulcers and 26.4% with aphthous ulcers only. In the UC subcohort, 48.6% of patients had an endoscopic Mayo score of 3, 40.3% had a score of 2, and 8.3% had a score of 1 at baseline (Table 3).

### 3.2. Stratification According to CDST

In CD patients, 22.8% were retrospectively classified into the low-response probability group (CDST = 1), 49.1% into the intermediate group (CDST = 2), and 28.1% into the high-response group (CDST = 3). In UC patients, 16.7% fell into the low (CDST = 1), 56.9% into the intermediate (CDST = 2), and 26.4% into the high-response (CDST = 3) probability groups.

### 3.3. Clinical Remission and Corticosteroid-Free Remission

**Figure 2:**
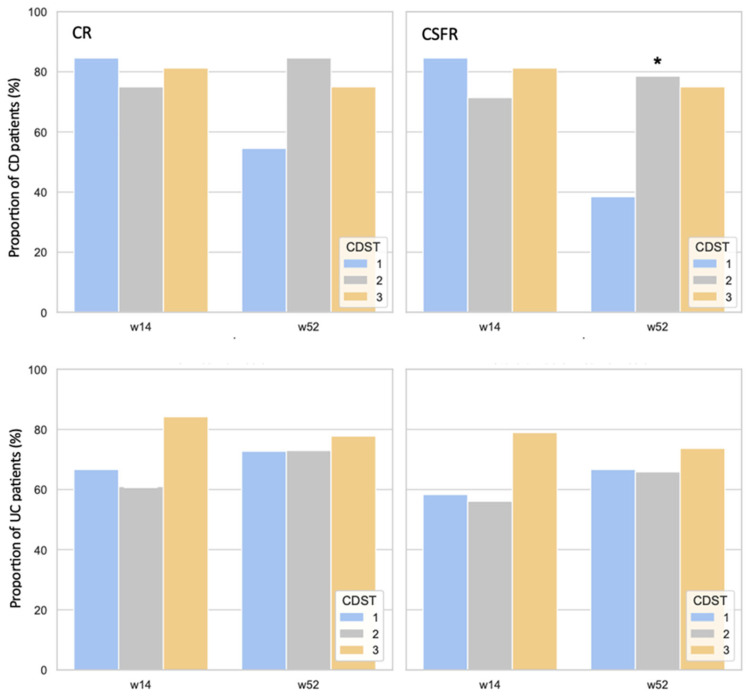
CR and CSFR in CD and UC patients according to CDST at weeks 14 and 52. CR = clinical remission; CSFR = corticosteroid-free remission; w = week; CDST = clinical decision support tool. * *p* < 0.05 according to Chi square test.

In the CD cohort, at week 14 all three CDST response groups showed high prevalences of CR (84.6%, 75.0%, and 81.2%, respectively) and CSFR (84.6%, 71.4%, and 81.2%, respectively), with no significant differences observed between groups. By week 52, however, the pattern appeared different: CR prevalences in the low-, intermediate-, and high-probability CDST groups were 54.5%, 84.6%, and 75.0%, respectively, and CSFR prevalences were 38.5%, 78.5%, and 75.0%, respectively. Although the lower prevalence of CR in the low-probability group compared with the intermediate- and high-probability groups did not reach statistical significance, the differences in CSFR between CDST response groups were statistically significant (*p* = 0.04; Figure 2).

In UC, surprisingly, the proportions of patients attaining CR and CSFR were comparable across the three stratification groups. At week 14, the prevalence of CR was 66.7%, 61.0%, and 84.2%, and the prevalence of CSFR was 58.3%, 56.1%, and 78.9% in the low-, intermediate-, and high-probability groups, respectively. At week 52, the prevalence of CR in the low- and intermediate-probability groups increased slightly over time to 72.7% and 72.3%, whereas in the high-probability group it was slightly lower, at 77.8%. A similar pattern was observed for CSFR, with prevalences of 66.7%, 65.9%, and 73.7% across the probability groups. No statistically significant associations were found between the primary outcomes and the stratification groups in UC (Figure 2).

### 3.4. Endoscopic Outcomes

**Figure 3:**
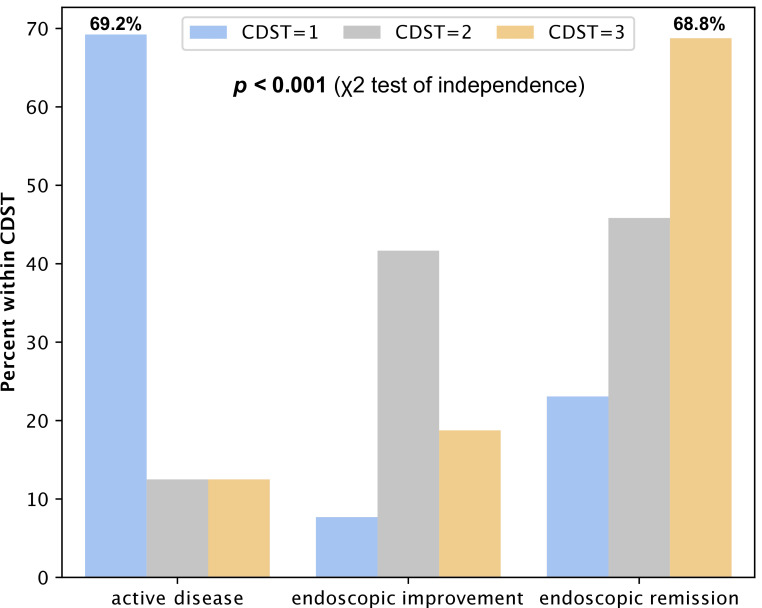
Percentage of CD patients within CDST groups according to endoscopic activity at follow-up endoscopy.

In CD patients, a statistically significant association (*p* < 0.001) was observed between CDST groups and endoscopic outcomes. The majority (69.2%) of patients in the low-probability group showed persistent EA at follow-up, whereas 68.8% in the high-probability group achieved ER (Figure 3). In intermediate-response patients, we observed only 12.5% of EA, 41.6% demonstrated EI and 45.8% ER.

**Figure 4:**
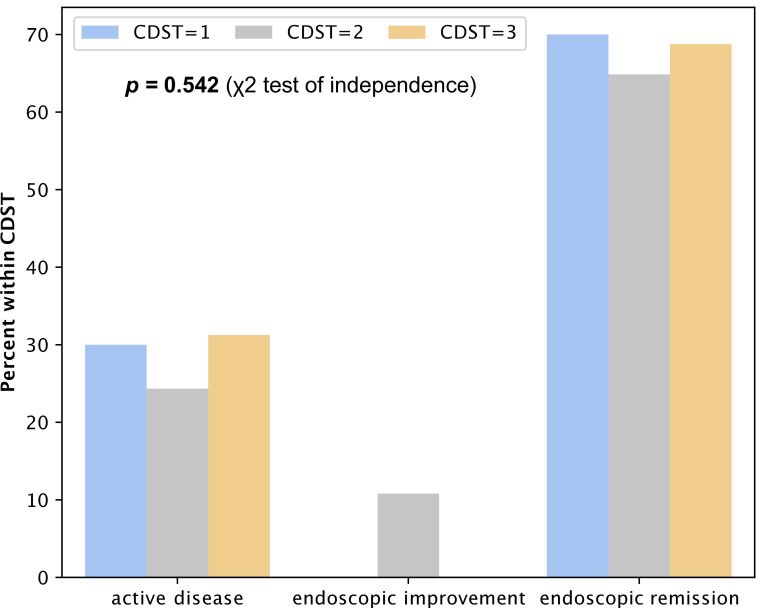
Percentage of UC patients within CDST groups according to endoscopic activity at follow-up endoscopy.

In UC patients, we observed no association between CDST stratification and endoscopic outcomes at follow-up (*p* = 0.542), with a high proportion of patients being in ER (70%, 64.8%, 68.8%) in all CDST groups, respectively (Figure 4).

To evaluate how well the selected clinical endpoint reflects objective disease control, we assessed the concordance between CR defined by PRO-2 and follow-up endoscopic outcomes at week 52. The results of this analysis are presented in the Supplementary Table S2. In patients with UC, the association was statistically significant (*p* = 0.01), whereas in CD a trend toward an association was observed (*p* < 0.07).

### 3.5. Treatment Persistence

In the CD subcohort, 19 patients (33.3%) discontinued vedolizumab (VDZ) during the follow-up period. The reasons for discontinuation were primary non-response in nine patients (47.4%), secondary loss of response in six (31.6%), adverse events in one (5.3%), and other reasons in three (15.8%). In the UC subcohort, 28 patients (38.9%) discontinued VDZ during follow-up. The reasons for discontinuation included primary non-response in 10 patients (35.7%), secondary loss of response in 12 (42.9%), adverse events in 1 (3.6%), and other reasons in 5 (17.9%).During the entire follow-up period, VDZ dose optimization with interval shortening to every 4 weeks was required in 13 CD patients (34.2%) and 10 UC patients (22.7%).

**Figure 5:**
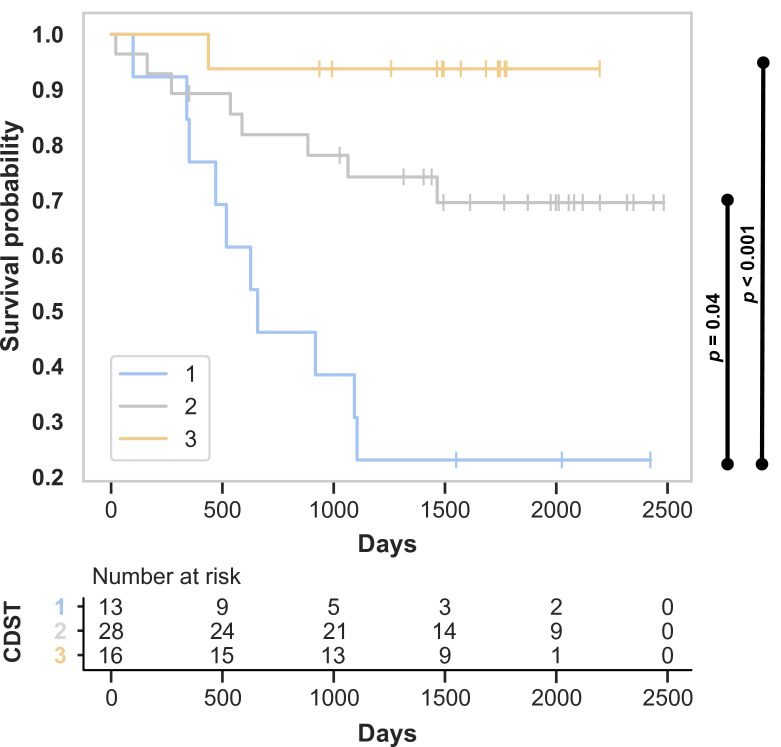
Treatment persistence for VDZ in CD patients according to CDST groups.

A notable finding was that treatment persistence at week 52 was significantly higher in CD patients classified into intermediate- and high-response groups compared to the low-response group (*p* = 0.004 and *p* < 0.001, respectively). Specifically, VDZ discontinuation rates were 76.9%, 28.6%, and 6.3% in the low-, intermediate-, and high-response groups, respectively (Figure 5).

**Figure 6:**
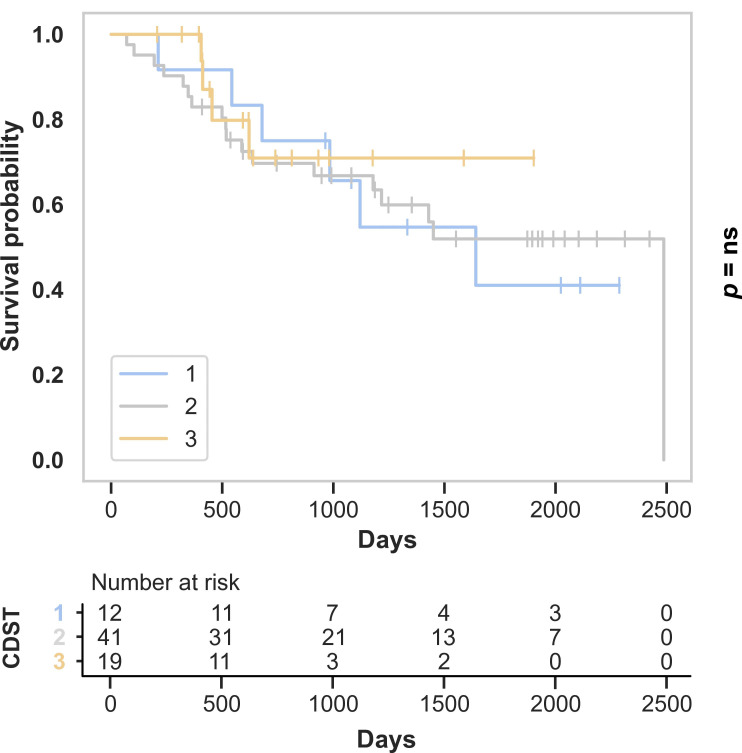
Treatment persistence for VDZ in UC patients according to CDST groups.

In UC, no statistically significant differences in treatment persistence were observed across stratification groups (*p* = 0.127). Discontinuation rates were 50.0% in low-, 34.9% in intermediate-, and 21.9% in high-probability groups (Figure 6).

## 4. Discussion

The broad range of IBD treatment options, while beneficial for patients, complicates clinical decision-making. Therefore, predicting treatment responders in advance is of essence. In this single-centre, retrospective study, we evaluated the utility of the VDZ-CDST as a pre-treatment predictive tool for clinical and endoscopic outcomes in a real-world, registry-based cohort of patients with IBD. The median disease duration was 14 years for CD and 8 years UC. Our results showed that the CDST was significantly associated with CSFR at week 52 (*p* = 0.04), endoscopic activity at follow-up (*p* < 0.001), and overall treatment persistence in CD (*p* < 0.001). In contrast, the CDST did not demonstrate predictive value for CR in CD, and none of the evaluated outcomes were associated with CDST in UC patients.

Ambiguous results of our study regarding the prediction of the primary outcomes, on one hand showing association of CSFR at week 52 in CD and on the other showing no association with CR as well as total lack of associations of CDST in UC, are in contrast with the original and validation studies by Dulai et al. and other authors [9,10,12,14]. One explanation for this discrepancy appears to relate to the definition of CR. In our study, CR was assessed exclusively via PRO-2, providing a much-simplified measure, whereas the original studies employed comprehensive indices such as the Crohn’s Disease Activity Index (CDAI) and Mayo scores, which are more nuanced and have higher sensitivity for disease activity [15,16,17,18]. On the other hand, our results support the ability of the CDST to predict endoscopic outcomes in CD, aligning with the validation cohort from the Victory consortium, where mucosal healing was defined similarly to endoscopic remission [12]. Given that mucosal healing correlates strongly with long-term prognosis, we find that this predictive aspect is particularly valuable for clinical decision-making [19,20].

Regarding CDST performance in UC, we observed relatively high remission rates despite CDST-based stratification. Namely rates of CSFR in UC were 66.7%, 65.9%, and 73.7% across probability groups at week 52. Moreover, we report high rates (70%, 64.8%, 68.8%, respectively) of ER across CDST groups as well. One reason for this apparent »failure« of CDST might be the low number of patients in our cohort or retrospective nature of it, since ER and CSFR data were collected retrospectively. It is also possible that the variables included in the CDST for UC do not fully reflect the underlying pathophysiology of UC, which may partially explain the absence of significant associations [21,22,23,24,25].

Treatment persistence, a proxy for long-term efficacy and tolerability, was significantly higher in CD patients within the intermediate and high-response groups, indicating that the CDST can assist in identifying patients likely to maintain therapy over time. This is consistent with the results of other studies [26,27,28,29,30]. In contrast, no such relationship was evident in UC in our study, suggesting differing predictive factors or disease dynamics. More importantly, treatment persistence in UC remained high over time also in the low-probability group according to CDST.

Accurate prediction of the disease course and therapeutic response is essential for optimizing management strategies in patients with IBD. Beyond existing CDSTs, machine learning—a subset of artificial intelligence (AI)—has shown promising results in analyzing complex clinical and molecular data to predict response to biologic therapies such as infliximab, VDZ, and ustekinumab in both CD and UC. In the future, AI-based approaches may therefore represent an important extension of current decision-support systems, enabling more precise treatment selection and improved resource utilization [31].

This study has several limitations. The most important is its retrospective design. Although we included all patients receiving VDZ who met the eligibility criteria, subgroup analyses by disease type and CDST category resulted in relatively small sample size. This limited statistical power and may have reduced the precision of effect estimates. Given the retrospective design, some degree of non-differential misclassification of exposure (CDST categorization) is possible. This would be expected to bias associations toward the null. However, because the CDST is a structured score derived from routinely collected clinical data, substantial misclassification is less likely. In addition, endoscopic outcomes were based on local assessment without central review, raising the possibility of outcome misclassification. If misclassification differed across CDST groups, the effect estimates could have been biassed in either direction. If the misclassification was non-differential, any true association may have been attenuated. This is particularly relevant for outcomes in which no significant association was observed. Luckily the endoscopic evaluation was done by one person, so the misclassification bias is less likely.

Despite these limitations, our study has several strengths. It reflects real-world practice through inclusion of a consecutive clinical cohort. Follow-up period was 1 year and the study incorporates not just clinical but also endoscopic outcomes, which are clinically and prognostically meaningful long-term treatment goals. Evaluating these outcomes therefore allows for a pragmatic and comprehensive assessment of the CDST’s utility in routine care. Additionally, we evaluated ability of CDST to predict treatment persistence with VDZ and were able to confirm its utility in CD. Prospective, multicentre studies with larger cohorts are needed to validate the predictive performance of the VDZ-CDST across diverse patient populations. Further refinement and external validation of this tool may support more individualized treatment decision-making and outcome-oriented management strategies in IBD.

## 5. Conclusions

VDZ-CDST–based response probability groups were associated with CSFR prevalence, endoscopic remission rates, and treatment persistence in patients with Crohn’s disease, suggesting that the score may serve as a supportive tool for therapeutic decision-making in this population. In contrast, these associations were not observed in ulcerative colitis, where clinical and endoscopic remission rates were relatively high across all CDST categories. These findings highlight the complexity of disease assessment in IBD and the importance of integrating objective markers into routine clinical practice.

## Figures and Tables

**Table 1:** CDST variables for Crohn’s disease [10].

No prior bowel surgery	+2 points
No prior TNFα inhibitors therapy	+3 points
No prior fistulising disease	+2 points
Baseline albumin	+0.4 points per g/L
Baseline CRP	−0.5 points if 3.0–10.0 mg/L −3.0 points if >10 mg/L

TNF = tumour necrosis factor, CRP = C-reactive protein.

**Table 2:** CDST variables for ulcerative colitis [9].

Disease duration ≥ 2 years	+3 points
No prior TNFα inhibitors therapy	+3 points
Baseline endoscopy moderate activity	+2 points
Baseline albumin	+0.65 points per g/L

TNF = tumour necrosis factor.

**Table 3:** Demographic data, disease and treatment characteristics.

	CD (N = 57)	UC (N = 72)
Gender (male; n, %)	22 (38.6%)	43 (59.7%)
Age at diagnosis (years), median (IQR)	33.9 (26.1)	32.1 (26.5)
Disease duration (years), median (IQR)	14.2 (17.3)	7.9 (11.6)
Disease location (Montreal)	L1; n = 11 (19.3%) L2; n = 13 (22.8%) L3; n = 19 (33.3%) +L4; n = 14 (24.6%)	E1; n = 0 (0%) E2; n = 26 (36.1%) E3; n = 46 (63.9%)
Fistulizing disease (n, %)	19 (33.3%)	-
Prior surgery	32 (56.1%)	2 (2.8%)
Concomitant CS therapy at baseline (n, %)	8 (14%)	25 (34.7%)
Concomitant IM therapy at baseline (n, %)	4 (7%)	8 (11.1%)
Prior anti-TNFα exposure (n, %)	33 (57.9%)	38 (52.8%)
Prior exposure to other AT		
Ustekinumab (n, %)	2 (3.5%)	0
JAK-i (n, %)	0	3 (4.2%)
Baseline CRP (mg/L), median (IQR)	6.0 (12.0)	5.0 (9.0)
Baseline albumin (g/L), median (IQR)	37.0 (5.0)	38.2 (4.4)
Baseline endoscopy in CD	N = 53	
1-Active disease with deep ulcers	37 (69.8%)	-
2-Active disease with aphthous ulcers	14 (26.4%)	-
3-Remission	2 (3.8%)	-
Baseline endoscopy in UC		
eMayo score 3	-	35 (48.6%)
eMayo score 2	-	29 (40.3%)
eMayo score 1	-	6 (8.3%)
eMayo score 0	-	2 (2.8%)
Probability of response to VDZ		
Low (n, %) (CDST group = 1)	n = 13; 22.8%	n = 12; 16.7%
Medium (n, %) (CDST group = 2)	n = 28; 49.1%	n = 41; 56.9%
High (n, %) (CDST group = 3)	n = 16; 28.1%	n = 19; 26.4%
Follow-up time (years), median (IQR)	4.7 (1.7)	3.2 (3.4)
VDZ treatment discontinuation		
No (n, %)	38 (66.7%)	44 (61.1%)
Yes (n, %)	19 (33.3%)	28 (38.9%)
Reason for treatment discontinuation	N = 19	N = 28
Primary non-response (n, %)	9 (47.4%)	10 (35.7%)
Secondary loss of response (n, %)	6 (31.6%)	12 (42.9%)
Adverse event (n, %)	1 (5.3%	1 (3.6%)
Other (n, %)	3 (15.8%)	5 (17.9%)
Dosing optimization among patients continuing VDZ treatment	N = 38	N = 44
No (n, %)	25 (65.8%)	34 (77.3%)
Yes (n, %)	13 (34.2%)	10 (22.7%)

CD = Crohn’s disease, UC = ulcerative colitis, n = number, CS = corticosteroid, IM = immunomodulator; TNFα = tumour necrosis factor alpha, AT = advanced therapy, JAK-i = Janus kinase inhibitor, CRP = C-reactive protein, eMayo = endoscopic Mayo, VDZ = vedolizumab, CDST = clinical decision support tool.

## Data Availability

The raw data supporting the conclusions of this article will be made available by the authors on reasonable request.

## Supplementary Materials

The following supporting information can be downloaded at: https://www.mdpi.com/article/10.3390/medicina62040722/s1, STROBE Statement—Checklist of items that should be included in reports of cohort studies; Table S1: Comparison of baseline characteristics of included and patients that were exluded due to missing data; Table S2: Concordance between CR according to PRO-2 and follow-up endoscopy at week 52.

## Abbreviations

The following abbreviations are used in this manuscript:

VDZ	Vedolizumab
CDST	Clinical decision support tool
IBD	Inflammatory bowel disease
CD	Crohn’s disease
UC	Ulcerative colitis
CR	Clinical remission
CSFR	Corticosteroid-free remission
EA	Endoscopic activity
EI	Endoscopic improvement
ER	Endoscopic remission
PRO	Patient reported outcome
CRP	C-reactive protein
TNFα	Tumour necrosis factor alpha
IQR	Interquartile range
CDAI	Crohn’s disease activity index
UR-CARE	United registries for clinical assessment and research
